# Heat-Related Kidney Injury Precedes Estimated GFR Decline in Workers at Risk of CKD

**DOI:** 10.1016/j.ekir.2024.11.1369

**Published:** 2024-12-07

**Authors:** Erik Hansson, Jason R. Glaser, Catharina Wesseling, Kristina Jakobsson, Nathan H. Raines, Ilana Weiss, Daniel Smith, Michael Silva-Peñaherrera, Rebekah A.I. Lucas, Paul Callejas, Denis Chavarria, David H. Wegman

**Affiliations:** 1 Occupational and Environmental Medicine, School of Public Health and Community Medicine, University of Gothenburg, Gothenburg, Sweden; 2 La Isla Network, Washington DC, USA; 3 Division of Nephrology, Department of Medicine, Beth Israel Deaconess Medical Center, Harvard Medical School, Boston, Massachusetts, USA; 4 School of Nursing, University at Buffalo, Buffalo, New York, USA; 5 School of Sport, Exercise and Rehabilitation Sciences, University of Birmingham, Birmingham, UK; 6 Occupational Health Management, Ingenio San Antonio/Nicaragua Sugar Estates Limited, Chichigalpa, Nicaragua; 7 Department of Public Health, University of Massachusetts Lowell, Lowell, Massachusetts, USA

## Introduction

Mesoamerican workers performing physically strenuous work^1^ are at high risk for chronic kidney disease of nontraditional origin (CKDnt). These workers also risk acute kidney injury (AKI), manifesting as either clinical AKI requiring health care encounters^2^^,^^3^; or as more subacute increases in serum creatinine (sCr) over a defined observation period, henceforth designated “incident kidney injury (IKI)”. In Mesoamerica, AKI and IKI have been linked to occupational heat stress; it is common among those performing intense, unshaded, manual labor in heat,^1^^,^^2^^,^^4^, ^5^, ^6^, ^7^, ^8^^,^S1–S4 and decreases with increasing rest-shade-hydration interventions.^2^^,^^6^, ^7^, ^8^

To investigate the relationship between AKI or IKI and CKDnt, we compared estimated glomerular filtration rate (eGFR) trends in sugarcane workers with AKI or IKI, with eGFR in sugarcane workers without evidence of comparable injury, at a large Nicaraguan sugarcane mill situated in a CKDnt hotspot.^9^ This mill excludes workers with elevated sCr during preemployment screening, and provides free health care, including AKI treatment to its employees. Many workers at the mill perform heavy physical work,S3 in wet-bulb globe temperatures often exceeding 30 °C,^2^ resulting in substantial heat stress.

Using mill hospital records, we identified all clinical AKI cases^2^ and selected those who met the Kidney Disease Improving Global Outcomes AKI criteriaS5 by comparing hospital sCr with preharvest sCr in male workers between November 2018 and October 2022 (AKI_case_ group). We extracted their postinjury sCr values and information about whether each case was permitted to return to work. Using data from our previously described rest-shade-hydration intervention study at the same sugarcane mill, the Adelante/PREP studies,^4^ across 4 harvest seasons (denoted H1–H4, encompassing 2017–2018 to 2020–2021), we identified IKI among burned and seed cane cutters during H1 and H2. In this study, we defined IKI as an increase in sCr of ≥0.3 mg/dlS5 over a 6-month harvest season^4^ (IKI_case_ group). Comparison groups were drawn from participants in the rest-shade-hydration intervention study between H1 and H4. The AKI_comparison_ group included all male workers with ≥2 preharvest eGFR measurements and the IKI_comparison_ group included male cutters who did not fulfill IKI criteria. Group selection is shown in Supplementary Figure S1.

We determined the baseline eGFR as the latest preinjury (for cases) and initial in cohort (comparison) and determined the average difference between baseline and follow-up preharvest eGFR values (Supplementary Methods).

## Results

Forty-six cutters fulfilled the IKI_case_ criteria, whereas 361 did not and formed the IKI_comparison_ group (Supplementary Figure S1). Out of 422 recorded clinical AKI cases between November 2018 and August 2023 at the company hospital, there were 179 fulfilling the criteria for the AKI_case_ group. These were compared with 1032 workers in the AKI_comparison_ group.

**Figure 1:**
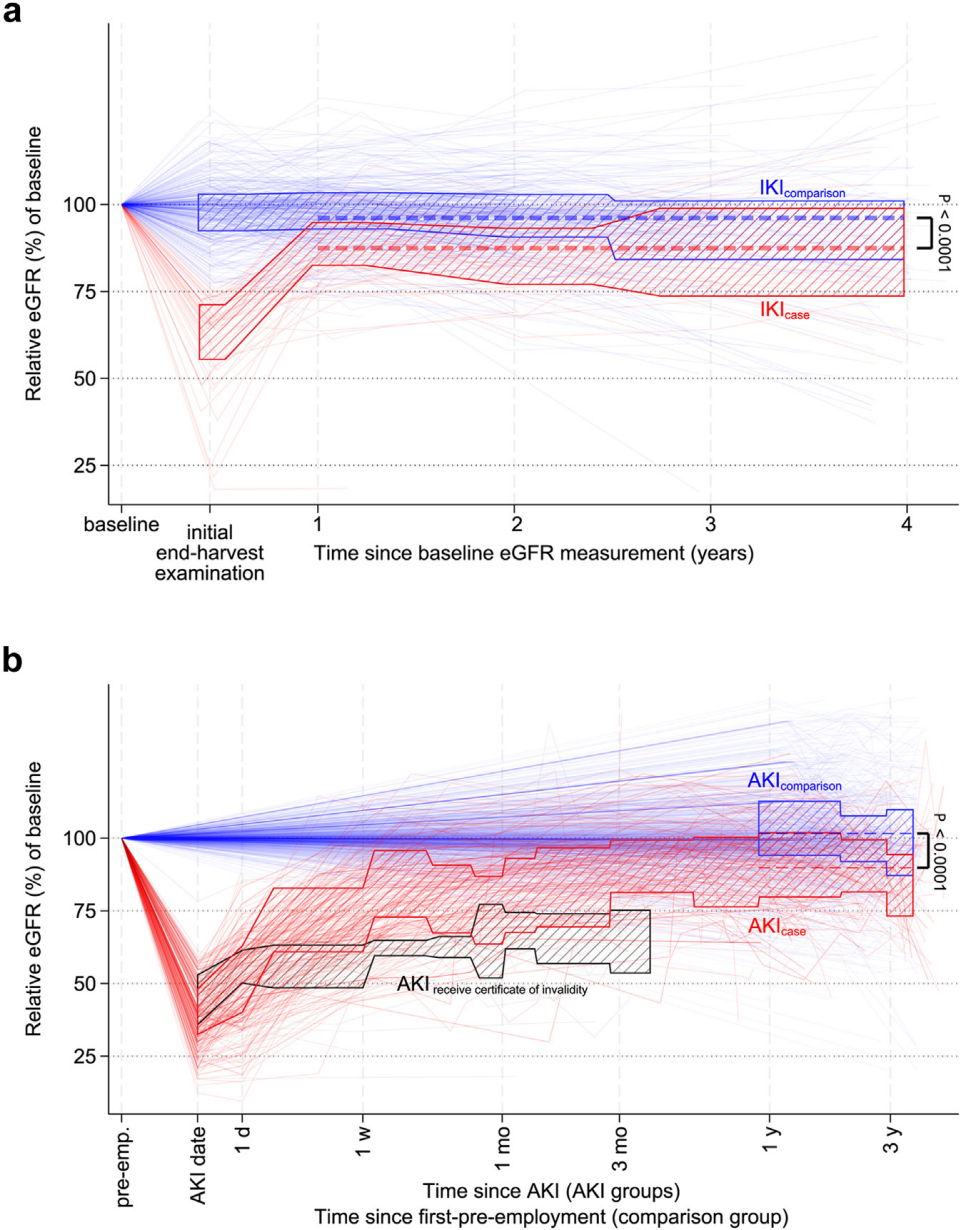
eGFR trajectories in workers with (a) incident kidney injury (IKI) and (b) clinically diagnosed acute kidney injury (AKI) along with comparison groups. Red denotes workers who developed kidney injury, blue denotes workers in comparison cohorts, and gray (b only) denotes workers with AKI who received a certificate of invalidity preventing further follow-up. Baseline eGFR was defined as the pre-harvest eGFR preceding IKI or AKI development for the IKI_case_ and AKI_case_ groups respectively, the earliest available pre-harvest eGFR meeting inclusion criteria for IKI_comparison_ and AKI_comparison_ groups. The shaded areas represent the interquartile range of the relative eGFR% at the corresponding time points compared with baseline. Thick dashed lines indicate the mean relative eGFR% for the IKI_case_ versus IKI_comparison_ (A) and AKI_case_ versus AKI_comparison_ groups (B). Thin solid lines indicate individual workers’ eGFR% trajectories. Observations demonstrating an increase in eGFR > 50% from baseline (Panel A, *n* = 16; Panel B, *n* = 39) are not shown but were included in the analyses. eGFR, estimated glomerular filtration rate.

Case and comparison groups respectively had similar ages and initial eGFRs and were followed-up for similar durations (Table 1). Compared with baseline eGFR, average eGFR during the follow-up period was lower in the IKI_case_ group (median difference [interquartile range]: −10.3 [−17.2 to −2.6] ml/min per 1.73 m^2^) than in the IKI_comparison_ group (median difference [interquartile range]: −3.3 [−10.2 to 1.6] ml/min per 1.73 m^2^; Table 1, Figure 1, Supplementary Figure S2). Similarly, it was lower in the AKI_case_ group (median difference [interquartile range]: −12.4 [−22.2 to −2.8] ml/min per 1.73 m^2^) than in the AKI_comparison_ group (median difference [interquartile range]: −0.6 [−8.0 to 9.5] ml/min per 1.73 m^2^; Table 1, Figure 1, Supplementary Figure S3).

**Table 1:** Descriptive data for sugarcane workers with kidney injury and corresponding comparison populations

Variable	Burned cane and seed cutters			ISA field workers		
	IKI_comparison_	IKI_case_	*P*-value^a^	AKI_comparison_	AKI_case_	*P*-value^a^
	361	46		1032	179	
Age at first visit in study, yrs, median (IQR)	28 (24–34)	27 (24–31)		28 (24–34)	27 (23–33)	
Initial eGFR, ml/min per 1.73m^2^, median (IQR)	109 (88–121)	98 (81–112)		110 (92–123)	106 (93–120)	
NSAID use, n (%)	57 (16%)	14 (30%)		119 (13%)^b^	6 (3%)	
Follow-up time during study, yrs, median (IQR)	3.0 (2.0–3.8)	3.0 (2.0–3.8)		2.2 (1.8–3.2)	1.8 (1.5–2.6)	
Number of follow-ups pre-harvest eGFR, median (IQR)	2 (2–3)	2 (2–2)		2 (1–2)	2 (1–4)	
Proportion with >1 follow-up pre-harvest eGFR (%)	81%	83%		60%	65%	
ΔeGFR%, median (IQR)	−2.9% (−9.4 to 1.9%)	−11.4% (−19.5% to −3.0%)	<0.001	−0.5% (−7.1% to 9.4%)	−11.4% (−19.9% to −2.6%)	<0.001
ΔeGFR, median (IQR) ml/min per 1.73 m^2^	−3.3 (−10.2 to 1.6)	−10.3 (−17.2 to −2.6)	<0.001	−0.6 (−8.0 to 9.5)	−12.4 (−22.2 to −2.8)	<0.001
Repeated measurement of eGFR > −5 ml/min per 1.73 m^2^ from baseline eGFR at any point during follow-up, n (%)	276 (76%)	22 (48%)	<0.001	783 (76%)	86 (48%)	<0.001

AKI, acute kidney injury; eGFR, estimated glomerular filtration rate; IKI, incident kidney injury; IQR, interquartile range; ISA, Ingenio San Antonio; NSAID, nonsteroidal anti-inflammatory drug; ΔeGFR, change in estimated glomerular filtration rate from initial pre-harvest eGFR to follow-up preharvest eGFR occurring >3 months after AKI and >11 months after initial pre-harvest eGFR (for AKI cases), or >11 months after initial preharvest eGFR (general workforce).

a From *t* test of difference in means.

b The response from the first postharvest was used and was available for 89% of all AKI_comparison_ workers.

Only 48% of the IKI_case_ and AKI_case_ groups recovered to an eGFR within 5 ml/min per 1.73 m^2^ of their baseline eGFR, whereas 76% of the IKI_comparison_ and AKI_comparison_ groups maintained an eGFR within 5 ml/min per 1.73 m^2^ during follow-up (Table 1). Adjusting for age or self-reported nonsteroidal anti-inflammatory drug use did not influence the results meaningfully (Supplementary Table S1).

In addition to the 179 AKI_case_ workers, there were 25 workers with AKI who were excluded from the follow-up because their poor initial recovery resulted in a certificate of invalidity and exclusion from the workforce. Of these, only 25% had recovered to within 75% of their baseline eGFR when post-AKI follow-up ceased, typically after 3 months (Figure 1b).

## Discussion

Kidney injury, requiring medical attention or detected via repeated sCr monitoring, was common among Nicaraguan sugarcane workers and associated with a lower eGFR persisting over several years. Such injuries may be an important cause of eGFR loss leading to CKDnt.

To our knowledge, this is the largest study with the longest post-injury follow-up period to investigate persistent loss of eGFR in a population at risk for CKDnt and the only study of its kind that includes comparison populations. We identified kidney injury in 2 distinct and complementary ways, with similar findings across both definitions. Participants with a kidney injury event on average lost approximately 10% of eGFR persistently, and about half never recovered to within 5 ml/min per 1.73 m^2^ of baseline eGFR. The eGFR decline occurred mainly as an incomplete recovery by the start of the next harvest that remained stably unrecovered in the workers who returned for subsequent pre harvest examinations (Figure 1). This magnitude of eGFR loss is biologically significant, particularly in this young and otherwise relatively healthy population who largely lack diabetes and hypertension.^3^^,^^4^

Our findings agree with previous studies showing that kidney injury arises among young male workers at risk for CKDnt when performing physically demanding work in hot environments,^1^, ^2^, ^3^, ^4^, ^5^, ^6^, ^7^, ^8^^,^S1–S4 and reinforce the connection between clinical and subclinical kidney injury events and CKDnt development.^4^^,^^5^^,^S6

Our study has important limitations which lead to a probable underestimation of effects. A healthy worker selection effect, causing unwell workers to leave the workplace more often, likely exists in this setting. A particularly strong type of healthy worker selection effect arose from the unavoidable exclusion of workers in the AKI_case_ group with the worst eGFR recovery, because they were not readmitted to the workforce and therefore did not have any pre-harvest sCr values during the follow-up period. We were unable to identify workers in the AKI_case_ group also participating in the Adelante/PREP cohort, so we did not exclude workers with IKI from the AKI_comparison_ group. We did not exclude workers with IKI in H3 or H4 from the IKI_comparison_ group. Workers in the AKI_case_ group may have been missed because the hospital only diagnosed those with sCr > 1.3 mg/dl with AKI. Workers with pre-harvest sCr < 1.0 mg/dl could have fulfilled the Kidney Disease Improving Global Outcomes criteria with a sCr < 1.3 mg/dl but would not have been diagnosed with AKI. Notably, some individuals in comparison groups developed a persistently lower eGFR. The inclusion of participants with unmeasured AKI in these comparison groups may be an explanation; however, AKI may not be an obligate feature of eGFR loss in CKDnt.

eGFR comparisons between IKI_case_ and IKI_comparison_, and AKI_case_ and AKI_comparison_ groups, respectively were done using creatinine values analyzed in parallel using the same instrument, meaning internal comparisons within the respective groups are valid. However, analytical variability may impact external comparisons of eGFR trends.

In conclusion, kidney injury arising from excessive occupational heat stress was associated with persistent nonrecovery of kidney function. Preventing kidney injury and providing adequate return-to-work guidance after injury might prevent CKDnt onset and progression.

## Disclosure

DC and PC worked for Ingenio San Antonio at the time the project was carried out, but the views presented here are not those of their employer. The authors designed and executed the study and have sole responsibility for the writing and content of the manuscript. All the other authors declared no competing interests.

## Supplementary Material

Supplementary File (PDF) **Supplementary Methods. Supplementary References. Figure S1.** Study flowsheet. **Figure S2.** eGFR trajectories in workers with incident kidney injury (IKI) along with comparison group. **Figure S3.** eGFR trajectories in workers with clinically diagnosed acute kidney injury (AKI) along with comparison group. **Table S1.** Linear regression of ΔeGFR% adjusting and not adjusting for age and NSAID use. **STROBE Checklist.**

## Footnotes

Supplementary File (PDF)

**Supplementary Methods**

**Supplementary References.**
**Figure S1.** Study flowsheet. **Figure S2.** eGFR trajectories in workers with incident kidney injury (IKI) along with comparison group. **Figure S3.** eGFR trajectories in workers with clinically diagnosed acute kidney injury (AKI) along with comparison group. **Table S1.** Linear regression of ΔeGFR% adjusting and not adjusting for age and NSAID use.
**STROBE Checklist.**
