# Supplementary material for: Heat-Related Kidney Injury Precedes Estimated GFR Decline in Workers at Risk of CKD
Source: Kidney Int Rep. 2024 Dec 7;10(3):948–51. doi: 10.1016/j.ekir.2024.11.1369 (PMC11993197; doi:10.1016/j.ekir.2024.11.1369)
Supplement: Supplementary File (PDF) — Supplementary Methods. Supplementary References. Figure S1. Study flowsheet. Figure S2. eGFR trajectories in workers with incident kidney injury (IKI) along with comparison group. Figure S3. eGFR trajectories in workers with clinically diagnosed acute kidney injury (AKI) along with comparison group. Table S1. Linear regression of ΔeGFR% adjusting and not adjusting for age and NSAID use. STROBE Checklist. [file mmc1.pdf]

Supplementary Material

Supplementary Methods

Setting and data sources

This study was conducted at Ingenio San Antonio (ISA), a sugarcane mill in a CKDnt hotspot in Northwestern Nicaragua [9]. A large proportion of ISA workers are recruited ahead of the harvest season that spans from November until April-June. A pre-employment health screen is required for employment and excludes workers based on kidney function criteria. Screening is repeated at least annually at ISA. Starting in the 2019-2020 harvest season, individuals were only hired if they had an eGFR >90 ml/min/1.73m^2 using the 2009 CKD-EPI equation [S7]. Prior to 2019, the hiring cut-off was sCr <1.3 mg/dl (for men) or <1.1 mg/dl (for women).

This study utilized two different data sources:

1. Mill records of pre-employment serum creatinine, AKI and post-AKI follow-up

Pre-employment sCr values from examinations obtained during the pre-harvest screen for all prospective workers were analyzed at the ISA hospital laboratory using a Roche Cobas C111 instrument and were kept in the ISA OSH department database.

A hospital on the ISA premises provides free healthcare for mill employees. AKI is diagnosed when symptomatic workers seeking hospital care are found to have a sCr of >1.3 mg/dl for men and >1.0 mg/dl for women [2]; Kidney Disease International Improving Global Outcomes (KDIGO) criteria of an increase of 0.3 mg/dl or 50% [S5] were not necessarily used for diagnosis at the hospital, but were additionally applied for this study. SCr is measured at the ISA hospital using the same Roche Cobas C111 instrument. For individuals diagnosed with AKI, the mill hospital provides care and performs laboratory examinations, and an occupational safety and health (OSH) nurse enters information on

demographic features, laboratory values, and other elements of medical history into a standardized form added to the ISA OSH medical record.

Some workers with AKI were permitted to return to work after monitoring their sCr trajectory. Post-AKI monitoring intervals and clearance to return to work were at the discretion of the mill hospital physician but clearance was generally based on recovery to below the 1.3 mg/dl sCr cut-off. Workers with AKI who did not recover were diagnosed with CKD and obtained a certificate of invalidity, ending their employment at ISA and entitling them to social benefits via the national health insurance system. The ISA OSH department has maintained a database on AKI diagnoses since January 2018, and on follow-up sCr values, clearance to return to work, and pre-harvest sCr values linked to AKI cases since November 2018.

2. Adelante/PREP study data

The Adelante/PREP study has been previously described [4, 6, 7, S3]. Briefly, this is a longitudinal study of sugarcane field workers conducted at ISA that investigates the efficacy of RSH interventions in this setting. In its initial year (2017-2018, henceforth H1), the study included only burned cane and seed cutters, irrigation repair workers, and field support staff linked to these workers. It expanded during the 2018-2019 harvest (H2) to also include workers involved in sugarcane seeding and weeding. Our current study includes data on pre- and end-harvest sCr values from the Adelante/PREP study, starting during the H1 harvest season and continuing during H2, the 2019-2020 harvest (H3), and the 2020-2021 harvest (H4). Because of drop-out during the harvest, end-harvest samples were missing from some workers. Between August and October 2021 (4-6 months after H4 ended), an effort was made to collect questionnaire data and serum and urine samples from all workers who were missing H4 end-harvest values and who had already provided samples at least twice during H1/H2. This sampling occasion is denoted the loss-to-follow-up sampling (LTFUS).

SCr for Adelante/PREP participants was measured at Lund University Hospital, Sweden (for samples collected H1-2 [6]) and the Costa Rican Agency for Biomedical Research (ACIB) (for samples collected

H3-4 and LTFUS) using Roche Cobas 701 and 6000 instruments, respectively. Samples from the same harvest were analyzed in the same batch. Serum samples for analyses were frozen following centrifugation at the ISA laboratory shortly after collection and kept at -80°C until analysis.

Ethics

Medical records data collection from the ISA OSH database was approved by the Comité de Ética para Investigaciones Biomédicas (CEIB) at Universidad Nacional Autónoma de Nicaragua (UNAN) León, Nicaragua (2019-114). Workers did not provide individual informed consent for the medical records review portion of this study. Data collection for the Adelante/PREP participants involved individual informed consent and was approved by the CEIB at UNAN León, Nicaragua (2017-47) and (2019-114). This study was additionally approved by the Swedish Ethical Review Authority (2023-01756-01).

Participant selection

To be eligible for this study all workers were required to have at least one initial (pre-injury for IKI and AKI groups) and one or more subsequent pre-harvest sCr measurement, which because of the pre-harvest sampling interval would be at least 1 year apart. We excluded female workers who constitute only 2% of IKI cases [6] and 5% of AKI cases [2]. 15% of the total ISA fieldwork workforce is female, including 0% of the burned cane cutters and 25% of the seed cutters. Study participant selection is shown in Figure S1.

Incident kidney injury (IKI) identified in active sugarcane cutters

The incident kidney injury (**IKI_{case}**) group included only burned cane and seed cutters participating in the Adelante/PREP study between H1 and H2 who developed a sCr increase of ≥0.3 mg/dl from their pre-harvest value to their end-harvest value during either harvest. Other work groups were not included as very few IKI cases occurred in these groups [6]. If a worker had pre- and end-harvest samples from both H1 and H2 but developed IKI only in H2, the H2 pre-harvest eGFR was considered

the baseline eGFR. We have previously shown that IKI was associated with elevated urine tubular injury markers and reduced hemoglobin and erythropoietin levels, thus indicating kidney injury, not only reduced glomerular filtration [S8]. Also, we have shown a high correlation between changes in eGFRs based on creatinine and on cystatin C across the harvest season [S9].

The IKI comparison (**$IKI_{comparison}$**) group included all burned cane and seed cutters in the Adelante/PREP study who had both pre- and end-harvest eGFR measurements in H1 or H2, and did not meet IKI criteria in either harvest. sCr results from the Lund or ACIB laboratory were used for eGFR calculation for both IKI_{case} and $IKI_{comparison}$ groups.

Acute kidney injury (AKI) diagnosed at hospital

For this study, the acute kidney injury (**"AKI_{case}"**) group included all male fieldworkers, regardless of job, treated at the ISA hospital for AKI between November 2018 and October 2022 and who had sCr values that met KDIGO AKI criteria of either a ≥0.3 mg/dl or ≥50% increase in sCr [S5] compared to their pre-harvest examination that year. Workers who were provided with a certificate of invalidity after AKI were excluded from the AKI_{case} group because very few of them (3/25) returned for pre-employment testing after receiving this certificate, and therefore were missing subsequent sCr values after their AKI event.

The **$AKI_{comparison}$** group included all male fieldworkers in the Adelante/PREP study between H1 and H4. SCr results from the ISA hospital laboratory were used for eGFR calculation for both AKI_{case} and $AKI_{comparison}$ groups.

Outcome definition

We used the 2021 CKD-EPI creatinine equation for all eGFR estimates [S10, S11]. We calculated change in eGFR for each individual by taking the difference between baseline and follow-up eGFRs. For the AKI_{case} and IKI_{case} groups, we defined baseline eGFR as the pre-harvest eGFR measured before the harvest during which they developed AKI or IKI, respectively. We defined follow-up eGFR as the

average of all pre-harvest eGFRs measured at least 3 months *after* their AKI or IKI event, as well as LTFUS values when available. We defined baseline eGFR as the earliest pre-harvest eGFR measurement for the $AKI_{comparison}$ group, and the earliest pre-harvest measurement from a harvest with both pre-and end-harvest values for the $IKI_{comparison}$ group. For comparison groups, we defined follow-up eGFR as the average of pre-harvest and LTFUS eGFR in measurements occurring after the baseline value. We expressed change in eGFR as both absolute (ΔeGFR) and relative changes (ΔeGFR%). We additionally categorized workers into those whose eGFR either did or did not recover to within 5 ml/min/1.73m^2 of their baseline eGFR at any point during follow-up. Only non-missing values were used for calculating ΔeGFR, no values were imputed for calculating ΔeGFR.

Statistical analysis

We used the Student's t-test to estimate the difference in ΔeGFR and ΔeGFR% between workers with AKI or IKI and their respective comparison groups. The proportions with and without a follow-up eGFR recovering to within 5 ml/min/1.73m^2 of their baseline were compared between groups using the chi-square test.

Linear regression was used to assess potential confounding by age and NSAID intake.

Stata version 18 was used for statistical analysis.

Supplementary References

S1.Gonzalez-Quiroz, M., Smpokou, E.T., Silverwood, R.J. , et al., *Decline in Kidney Function among Apparently Healthy Young Adults at Risk of Mesoamerican Nephropathy.* J Am Soc Nephrol, 2018. **29**(8): p. 2200-2212.

S2. Sorensen, C.J., Butler-Dawson, J., Dally, M., et al., *Risk Factors and Mechanisms Underlying Cross-shift Decline in Kidney Function in Guatemalan Sugarcane Workers.* J Occup Environ Med, 2018. 61(3):239-250.

S3. Lucas, R.A.I., Skinner, B.D. Arias-Monge, E., et al., *Targeting workload to ameliorate risk of heat stress in industrial sugarcane workers.* Scand J Work Environ Health, 2023. **49**(1): p. 43-52.

S4. Petropoulos, Z.E., Keogh, S.A., Jarquín, E., et al., *Heat stress and heat strain among outdoor workers in El Salvador and Nicaragua.* Journal of Exposure Science & Environmental Epidemiology, 2023. **33**(4): p. 622-630.

S5. Kidney Disease Improving Global Outcomes. *KDIGO Clinical Practice Guideline for Acute Kidney Injury*. 2012; Available from: https://kdigo.org/wp-content/uploads/2016/10/KDIGO-2012-AKI-Guideline-English.pdf.

S6. Fischer, R.S.B., Vangala, C. Mandayam, S., et al., *Clinical markers to predict progression from acute to chronic kidney disease in Mesoamerican nephropathy.* Kidney Int, 2018. **94**(6): p. 1205-1216.

S7. Levey, A.S., Eckardt, K.U., Dorman, N.M., et al., *A New Equation to Estimate Glomerular Filtration Rate.* Annals of Internal Medicine, 2009. **150**(9): p. 604-612.

S8. Hansson, E., Wegman, D.H., Wesseling, C., et al., *Markers of kidney tubular and interstitial injury and function among sugarcane workers with cross-harvest serum creatinine elevation.* Occupational and Environmental Medicine, 2021. 79(6):396-402.

S9. Andersson A, Hansson E, Ekström U, Grubb A, Abrahamson M, Jakobsson K, Xu Y.

Large difference but high correlation between creatinine and cystatin C

estimated glomerular filtration rate in Mesoamerican sugarcane cutters. Occup

Environ Med. 2022 Jul;79(7):497-502.

S10. Inker, L.A., Eneaya, N.D., Coresh, J., et al., *New Creatinine- and Cystatin C-Based Equations to Estimate GFR without Race.* N Engl J Med, 2021. **385**(19): p. 1737-1749.

S11. Raines, N.H., Inker, L.A., Seegmiller, J.C., et al., *Estimated Versus Measured Glomerular Filtration Rate in Men at Risk for Mesoamerican Nephropathy.* Am J Kidney Dis, 2023. **81**(3): p. 370-373.

Supplemental Results

Supplement Table 1. Linear regression of ΔeGFR% adjusting and not adjusting for age and NSAID use.

	IKI				AKI			
	Basic model		Adjusted model		Basic model		Adjusted model	
	β	p	β	p	β	p	β	p
Kidney injury	-8.7 (-13.2, -4.2)	<0.001	-8.5 (-13.1, -4.0)	<0.001	-11.8 (-14.7, -8.8)	<0.001	-11. 6 (-14.4, -8.8)	<0.001
NSAID			-0.9 (-4.7, 2.9)	0.66			-0.5 (-3.7, 2.8)	0.77
Age (years)			0.033 (-0.015, 0.022)	0.73			0.14 (0.01,0.26)	0.04

β = regression coefficient, % estimated glomerular filtration rate change from pre-injury to average post-injury. p = p-value

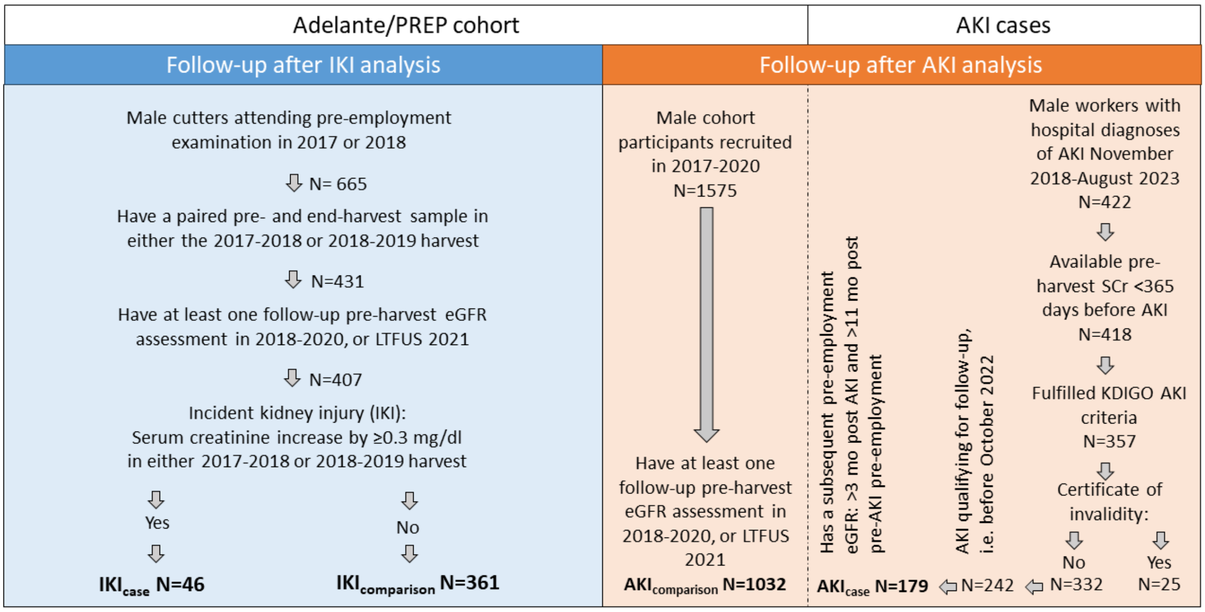

Supplement Figure S1. Study flowsheet. IKI, incident kidney injury, defined as a serum creatinine increase of ≥0.3 mg/dl from pre- to end-harvest sampling; AKI, acute kidney injury; KDIGO, Kidney Disease Improving Global Outcomes; LTFUS, loss-to-follow-up sampling.

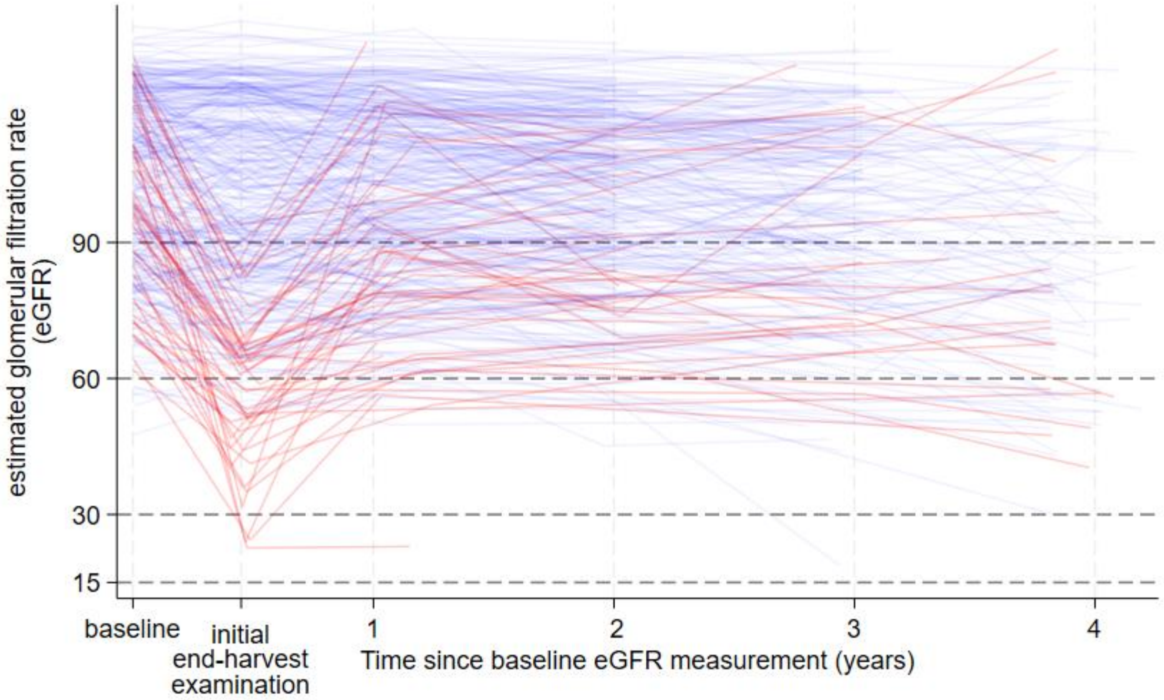

Supplement Figure S2 eGFR trajectories in workers with incident kidney injury (IKI) along with comparison group.

Red denotes workers who developed kidney injury, **blue** denotes comparison workers. Baseline eGFR was defined as the pre-harvest eGFR preceding IKI for the IKI_{case} group respectively and the earliest available pre-harvest eGFR meeting inclusion criteria for $IKI_{comparison}$.

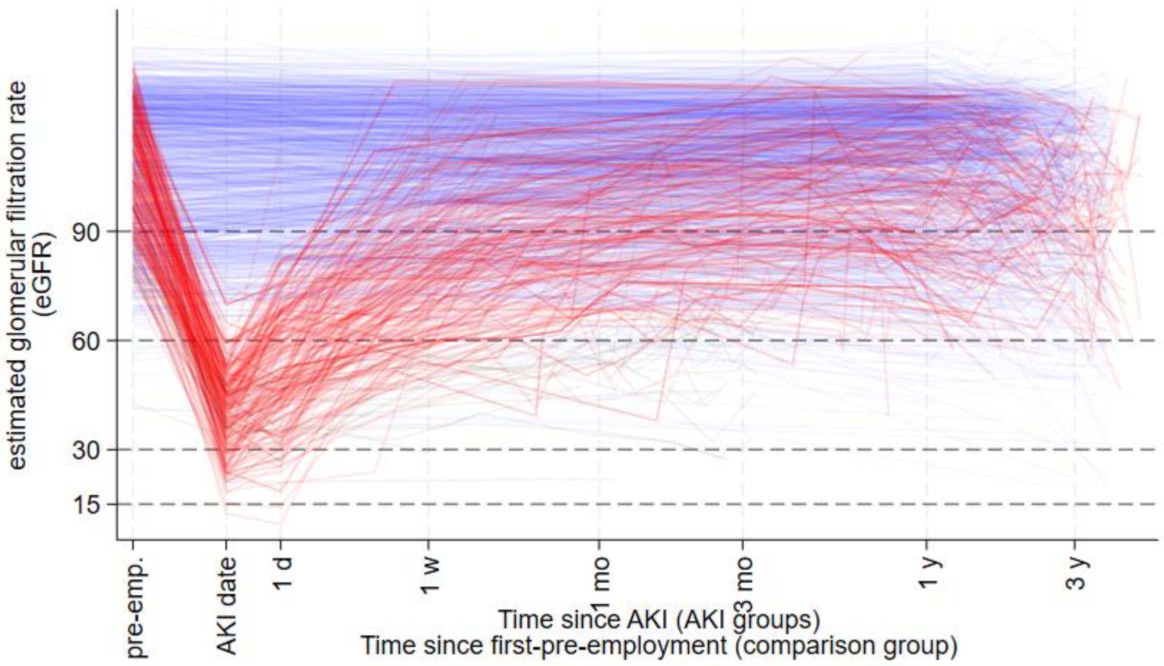

Figure S3 eGFR trajectories in workers with clinically diagnosed acute kidney injury (AKI) along with comparison group.

Red denotes workers who developed kidney injury, **blue** denotes comparison workers. Baseline eGFR was defined as the pre-harvest eGFR preceding IKI for the AKI_{case} group respectively and the earliest available pre-harvest eGFR meeting inclusion criteria for $AKI_{comparison}$.

STROBE Statement—checklist of items that should be included in reports of observational studies

	Item No	Recommendation	Page No
**Title and abstract**	1	(*a*) Indicate the study's design with a commonly used term in the title or the abstract	1
		(*b*) Provide in the abstract an informative and balanced summary of what was done and what was found	N/A
**Introduction**			
Background/rationale	2	Explain the scientific background and rationale for the investigation being reported	3
Objectives	3	State specific objectives, including any prespecified hypotheses	3-4
**Methods**			
Study design	4	Present key elements of study design early in the paper	3-4
Setting	5	Describe the setting, locations, and relevant dates, including periods of recruitment, exposure, follow-up, and data collection	3-4, S2-S4
Participants	6	(*a*) *Cohort study*—Give the eligibility criteria, and the sources and methods of selection of participants. Describe methods of follow-up<br>*Case-control study*—Give the eligibility criteria, and the sources and methods of case ascertainment and control selection. Give the rationale for the choice of cases and controls<br>*Cross-sectional study*—Give the eligibility criteria, and the sources and methods of selection of participants	S2-S6
		(*b*) *Cohort study*—For matched studies, give matching criteria and number of exposed and unexposed	

		Case-control study—For matched studies, give matching criteria and the number of controls per case	
Variables	7	Clearly define all outcomes, exposures, predictors, potential confounders, and effect modifiers. Give diagnostic criteria, if applicable	S4-S6
Data sources/ measurement	8*	For each variable of interest, give sources of data and details of methods of assessment (measurement). Describe comparability of assessment methods if there is more than one group	*S4-S6*
Bias	9	Describe any efforts to address potential sources of bias	S4-S6
Study size	10	Explain how the study size was arrived at	S4-S6
Quantitative variables	11	Explain how quantitative variables were handled in the analyses. If applicable, describe which groupings were chosen and why	S5-S6
Statistical methods	12	(*a*) Describe all statistical methods, including those used to control for confounding	S5-6
		(*b*) Describe any methods used to examine subgroups and interactions	NA
		(*c*) Explain how missing data were addressed	S6
		(*d*) *Cohort study*—If applicable, explain how loss to follow-up was addressed<br>*Case-control study*—If applicable, explain how matching of cases and controls was addressed<br>*Cross-sectional study*—If applicable, describe analytical methods taking account of sampling strategy	S4-S6
		(*e*) Describe any sensitivity analyses	S6

Continued on next page

Results

Participants	13*	(a) Report numbers of individuals at each stage of study—eg numbers potentially eligible, examined for eligibility, confirmed eligible, included in the study, completing follow-up, and analysed	Fig S1
		(b) Give reasons for non-participation at each stage	Fig S1.
		(c) Consider use of a flow diagram	Fig S1.
Descriptive data	14*	(a) Give characteristics of study participants (eg demographic, clinical, social) and information on exposures and potential confounders	Table 1
		(b) Indicate number of participants with missing data for each variable of interest	
		(c) *Cohort study*—Summarise follow-up time (eg, average and total amount)	Table 1
Outcome data	15*	*Cohort study*—Report numbers of outcome events or summary measures over time	Table 1, Fig 1, Figs. S2 and S3
		Case-control study—Report numbers in each exposure category, or summary measures of exposure	

		Cross-sectional study—Report numbers of outcome events or summary measures	
Main results	16	(*a*) Give unadjusted estimates and, if applicable, confounder-adjusted estimates and their precision (eg, 95% confidence interval). Make clear which confounders were adjusted for and why they were included	Table 1, 4
		(*b*) Report category boundaries when continuous variables were categorized	N/A
		(*c*) If relevant, consider translating estimates of relative risk into absolute risk for a meaningful time period	N/A
Other analyses	17	Report other analyses done—eg analyses of subgroups and interactions, and sensitivity analyses	4, Table S1
**Discussion**			
Key results	18	Summarise key results with reference to study objectives	5
Limitations	19	Discuss limitations of the study, taking into account sources of potential bias or imprecision. Discuss both direction and magnitude of any potential bias	5-6
Interpretation	20	Give a cautious overall interpretation of results considering objectives, limitations, multiplicity of analyses, results from similar studies, and other relevant evidence	5-6
Generalisability	21	Discuss the generalisability (external validity) of the study results	6
**Other information**			
Funding	22	Give the source of funding and the role of the funders for the present study and, if applicable, for the original study on which the present article is based	8
